# Nights, Bites, and Cries

**DOI:** 10.4269/ajtmh.21-0982

**Published:** 2022-09-12

**Authors:** Kaliaperumal Karthikeyan, M Prarthana

**Affiliations:** Department of Dermatology, Venereology & Leprosy, Sri Manakula Vinayakar Medical College & Hospital, Puducherry, India

The arrival of the monsoon is celebrated as a festival in Tamil Nadu, the southern part of India. The rain is believed to bring prosperity to the people whose livelihood is mainly dependent on agriculture. Farmers celebrate the onset of the monsoon as a gesture of gratitude, looking for an abundant yield by offering prayers at riverbanks, lakes, and ponds to become full by the rains. These are the main sources of water during the dry season. People celebrate by lighting lamps, praying to their deities, offering flowers, and floating lamps to the river goddess. The association of this ritual with prosperity is common, and the history of this practice dates back to ancient times. These festivals are commonly noticed in rural areas and villages situated on riverbanks, which is exceptional because it brings families, friends, and people together.

It is common during this season to find the outpatient clinic filled with many young women carrying their wailing children on their hips during and after the monsoon. One among them was Mrs. Lakshmi, an agricultural laborer who toiled in the sun every day to make her daily earnings. She was draped in a bright-yellow saree with a child clinging to her waist—her 3-year-old daughter Alli. Alli was hesitant to visit the hospital, which was obvious by her wails, which were silenced promptly by her mother with a stare.

Along with the incessant rain, the monsoon brings in dangerous vector-borne diseases, which are spread by the bites of mosquitoes such as *Aedes* and *Anopheles*. These insects breed in stagnant water and multiply in number. The mosquitoes and insects that come around during this season cause numerous types of insect bite reactions and papular urticaria. The number of cases rises with monsoon onset and are the result of waterlogging and clogging in domestic and public surroundings, as the sewage and drainage plants still need improvement in rural parts of India. Lack of personal hygiene, overcrowding, and poor sanitation are critical risk factors for these diseases. Various manifestations are seen, ranging from urticaria to multiple erythematous itchy papules in children who usually present with incessant cries at night. The winged nuisance is called *Sullan* in local vernacular, denoting the sharp stinging pain and itching that follows the mosquito bite.

Lakshmi complained that her daughter had multiple itchy eruptions and intense pruritus exacerbated by mosquito bites. I was relieved that she had no fever or signs of the dangerous vector-borne diseases. Lakshmi was more concerned about her daughter’s suffering at night and the incessant crying. We gathered the information from her that a few other children from her locality were also affected with similar issues. Most of the houses located in rural areas are thatched mud houses with a crude floor made of mud and cow-dung paste. They have a single room sufficient for storing their belongings. Hence, people generally choose to sleep on the mud floor outside their homes for better ventilation. Lakshmi explained that the mosquito bites are unavoidable in such circumstances because they do not cover themselves adequately and she sleeps outdoors with her children around her, exposed to the night sky, the rains, and the insects looking for their next blood meal. The mosquitoes target the children, and Alli was no exception. Continuous mosquito bites and the itching that follows, along with crying, worsens temperaments at night and leads the mother to lose sleep and miss work on more than a few occasions.

Aware of the source of the ailment, she explained about her helplessness and was worried, despite the ongoing local festival in their community. She was instructed about personal protection that could prevent her child from mosquito bites. Lakshmi explained that she was a daily laborer and, during rains, does not have a job. Her income per day is sufficient to suit only her day-to-day needs, making mosquito repellents or mosquito nets unaffordable.

Although vector control programs are in place, their execution has many gaps resulting from the lack of funds and increasing population rates. To exacerbate this situation, the general public is unaware of the breeding sources and the biology of mosquitoes.

The success of any treatment lies in the use of local resources in the management of disease. Because they are a cheap alternative and are easily accepted by the rural population, Lakshmi was instructed to wear long-sleeve clothes to avoid mosquito bites. In addition, rural areas have abundant neem trees, and the foliage is generally dumped as waste. I advised her that dried neem leaves could be used for fumigation by burning them in the evenings and early mornings. The smoke generated is dense and surrounds the perimeter, and is toxic to the mosquitoes. It can be done near the houses, and fumes will ward off the mosquitoes for quite some time. She told us that it was a common practice to burn the dry leaves in her locality as a method of waste disposal, but she did not know its significance. She was surprised to know the low-cost alternative and cursed herself for being unschooled and ignorant. She informed me they did not conduct this burning regularly, and confirmed that she would take care of the breeding sources in her domestic area by dry fumigation with neem leaves.

Unlike other infectious diseases, the problems caused by the march of mosquitoes is ever-expanding and relentless because of improper drainage, unplanned urbanization, and poor vector control. Addressing a complex disease requires government policies for a better infrastructure, sanitation facilities, vector control, and clinical management. However, scenarios such as this one necessitate lateral thinking and various indigenous, feasible ways to effect vector control and other measures. Other local practices, which are safe and available, include the topical application of neem oil, which has benefits against warding off mosquitoes.

During her subsequent visits, Lakshmi informed me that the mosquito menace has decreased in their domain. Through self-help groups, she mobilized several other females in joining her to abolish breeding sites and conduct dry fumigation. Literacy and knowledge are the only wealth that can uplift any society, and this case proves it. It is an appalling truth that most people of low socioeconomic status have learned to adapt their lifestyle and live despite all the odds stacked against them, including mosquitoes. Until this menace is controlled, the nights of many an infant will remain a nightmare.

